# A Cohort of 469 Mayer–Rokitansky–Küster–Hauser Syndrome Patients—Associated Malformations, Syndromes, and Heterogeneity of the Phenotype

**DOI:** 10.3390/jcm13020607

**Published:** 2024-01-21

**Authors:** Martin Pietzsch, Birgitt Schönfisch, Alice Höller, André Koch, Annette Staebler, Katharina Dreser, Kristina Bettecken, Lisa Schaak, Sara Yvonne Brucker, Katharina Rall

**Affiliations:** 1 Department of Obstetrics and Gynecology, University of Tübingen, 72076 Tübingen, Germany; martin.pietzsch@med.uni-tuebingen.de (M.P.); sara.brucker@med.uni-tuebingen.de (S.Y.B.); 2 Research Institute for Women’s Health, University of Tübingen, 72076 Tübingen, Germany; andre.koch@med.uni-tuebingen.de; 3 Department of Pathology, University of Tübingen, 72076 Tübingen, Germany

**Keywords:** Mayer–Rokitansky–Küster–Hauser syndrome, MRKHS, uterine rudiments, uterine aplasia, vaginal aplasia, endometrial tissue, Müllerian anomalies

## Abstract

The Mayer–Rokitansky–Küster–Hauser syndrome is characterized by aplasia of the uterus and upper two-thirds of the vagina. While it can appear as an isolated genital malformation, it is often associated with extragenital abnormalities, with little still known about the pathogenetic background. To provide an overview of associated malformations and syndromes as well as to examine possible ties between the rudimentary tissue and patient characteristics, we analyzed a cohort of 469 patients with MRKHS as well as 298 uterine rudiments removed during surgery. A total of 165 of our patients (35.2%) had associated malformations (MRKHS type II). Renal defects were the most common associated malformation followed by skeletal abnormalities. Several patients had atypical associated malformations or combined syndromes. Uterine rudiments were rarer in patients with associated malformations than in patients without them. Rudiment size ranged from 0.3 cm^3^ to 184.3 cm^3^ with a mean value of 7.9 cm^3^. Importantly, MRKHS subtype or concomitant malformations were associated with a different frequency of uterine tissue as well as a different rudiment size and incidence of endometrial tissue, thereby indicating a clear heterogeneity of the phenotype. Further research into the associated molecular pathways and potential differences between MRKHS subtypes is needed.

## 1. Introduction

The Mayer–Rokitansky–Küster–Hauser syndrome (MRKHS) is a congenital disorder of the Müllerian ducts, which typically results in the partial or complete aplasia of the uterus and the upper two-thirds of the vagina in women with a normal female karyotype [1]. As the ovaries remain hormonally functional, patients with MRKHS exhibit normal secondary sexual characteristics with regular pubarche and thelarche during puberty [2,3]. The syndrome has been estimated to occur in about 1 of 4500–5000 female live births, with only a few population-based studies of prevalence published [4,5]. Patients with MRKHS usually possess one or two uterine rudiments that most often consist of myometrium and occasionally also endometrial tissue [3,6]. Whilst the MRKHS can appear as an isolated genital malformation (type I), it is often associated with additional extragenital abnormalities that affect the kidneys (unilateral agenesis, ectopia) or skeleton (scoliosis, block vertebrae) and occasionally the auditory system, eyes, or heart (type II). The combination of uterovaginal aplasia with both renal and skeletal malformations has been established as a subgroup of type II and is called MURCS association (Müllerian, Renal and Cervicothoracic somite abnormalities) [2,7,8]. Furthermore, several syndromes or associations such as thrombocytopenia-absent radius (TAR) syndrome, Holt–Oram syndrome, or VACTERL (vertebral defects, anal atresia, cardiac defects, tracheoesophageal fistula/esophageal atresia, renal anomalies, and limb abnormalities) association have been reported in combination with MRKHS in some individuals [8,9,10,11]. Whilst European studies on MRKHS cohorts have shown associated malformations in 43.5–53.0% of all patients [5,7,8,12], the results of a Chinese study by Pan et al. (2016) hint towards geographic discrepancies with only 7.2% of all patients having extragenital malformations [13].

Due to several cases of familial clustering, a genetic cause for the MRKHS seems most likely. However, the exact etiology is still unknown [8,14]. Several chromosomal aberrations have been found in recent years, for instance affecting chromosomes 1, 4–5, 7–8, 10, 13, 16–17, 22, and X, which may be causal in a subset of patients [15,16,17,18,19]. Further mutations and possibly damaging variants have been identified on some occasions in developmental genes affecting Müllerian duct formation and elongation such as LHX1 [17,20,21], TBX6 [21,22,23], WNT9B [24,25], and WNT4 [26,27,28]. Moreover, network analysis of protein interaction data offers new possibilities to put detected copy number variants (CNVs) into a biological context, thereby showing how different genetic alterations found in patients might contribute to the pathophysiology of MRKHS [19]. 

However, a genetic cause has not yet been discovered for the vast majority of patients. Cases of discordant monozygotic twins [29,30,31] underline the importance of also looking for epigenetic factors or local dysfunctions such as impaired hormone receptors [6,32,33]. Therefore, the rudimentary tissue has come more into the focus of current research. Firstly, it allows analysis of genetic differences at the transcriptional level. Secondly, newly established cell culture models offer a unique opportunity to study the characteristics and capabilities of the diseased tissue [32,34,35,36]. Functional models such as patient-derived endometrial organoids may therefore allow new insights into the pathogenesis of the MRKHS [34,37]. However, rudimentary uterine tissue is difficult to obtain for researchers due to the invasive nature of its retrieval. Before our work, no study had examined possible ties between patient characteristics and the frequency, size, as well as structure of rudimentary uterine tissue in a large number of patients. 

Our study provides an overview of typical and atypical malformations as well as syndromes associated with MRKHS by analyzing the characteristics of 469 patients. As one of the few centers worldwide with access to rudimentary uterine tissue in significant numbers, we were also able to examine possible ties between patient characteristics, such as associated malformations and the presence of uterine rudiments, as well as rudiment size and frequency of endometrial tissue, to deepen our understanding of the diseased tissue itself. 

## 2. Materials and Methods

### 2.1. Patients

This study is a retrospective analysis of 469 patients who presented with MRKHS at the Department of Obstetrics and Gynecology at the University Hospital of Tübingen (Germany) between January 2014 and December 2022. A standardized anamnestic questionnaire was handed out to all these patients, most of them undergoing laparoscopy-assisted creation of a neovagina. Preoperative assessment typically included magnetic resonance imaging of the pelvis and renal systems together with a urography as well as renal ultrasonography. Examination of possible uterine rudiments and malformations was performed during laparoscopy. Every rudiment was assessed visually regarding the possible existence of intrauterine endometrium, based on size, color, and consistency. The uterine rudiments were removed if the patient had a history of cyclic lower abdominal pain, if intrauterine endometrium was suspected due to ultrasound or MRI, or if the patient wished prophylactic removal after consultation. Every rudiment received standardized histopathological examination including measurement of size. If no endometrial tissue was macroscopically visible, rudiments were randomly sampled. All patients were informed about the systematic analysis of data for research purposes and provided their written consent. This study was approved by the Ethics Committee of the University of Tübingen (protocol code 077/2022BO2). 

### 2.2. MURCS Association

Patients were classified as MURCS if the combination of uterine aplasia/hypoplasia, renal agenesis, and/or ectopy and vertebral anomalies due to cervicothoracic somite dysplasia was present.

### 2.3. VACTERL Association

For classification as VACTERL association, the diagnostic criteria proposed by van de Putte et al. (2019) were applied. Congenital anomalies that are considered to be part of the VACTERL spectrum were therefore categorized into major and minor VACTERL features. Please refer to van de Putte et al. (2019) [38] for a complete list of anomalies that count as VACTERL features.

### 2.4. Statistical Analysis 

Study data were collected and managed using REDCap electronic data capture tools hosted at the Department of Obstetrics and Gynecology at the University Hospital of Tübingen [39,40]. Data were depicted by the mean with standard deviation (SD) and/or median, or numbers and percentages using Microsoft Excel 2019 and GraphPad Prism 9 (GraphPad Holdings, LLC, San Diego, CA, USA). Percentages are based on the number of patients for whom the respective characteristic was recorded. The chi-square test was used for count data and Welch’s *t*-test for continuous variables. A significance level α of 0.05 was used for statistical analysis. 

## 3. Results

In our cohort of 469 patients, 304 patients (64.8%) presented with MRKHS type I. A total of 165 patients (35.2%) had associated malformations (type II), with 8 patients (1.7%) fulfilling the criteria for MURCS syndrome. 

Among the 165 patients with additional malformations, 107 (64.8%) had renal malformations, 81 (49.1%) had malformations of the skeleton, and 39 (23.6%) patients had other associated malformations—typical (see Table 1) and atypical ones (see Table 2). Combined renal and skeletal defects were present in 34 (20.6%) patients. Amidst the 107 patients with renal abnormalities, there were 68 cases of unilateral kidney agenesis, 18 pelvic kidneys, 9 malrotated kidneys, 14 duplex kidneys, and 6 horseshoe kidneys. Additional urological abnormalities, including ureteral strictures, were present in 11 patients. 

Of the 81 patients with skeletal malformations, the spine was most often affected with 45 cases of scoliosis and 10 cases of block vertebrae (including Klippel–Feil Syndrome). Five patients presented with Klippel–Feil syndrome, all of which also displayed renal malformations. Furthermore, there were 5 cases of polydactyly, 3 cases of syndactyly, 8 cases of hip dysplasia, and 3 patients with congenital talipes equinovarus (CTEV), as well as 22 patients with other skeletal anomalies. 

Of the 39 other typical and atypical associated malformations, auditory (6, 3.6%), ocular (2, 1.2%), and cardiac defects (18, 10.9%) were frequently found. Cardiac defects included tetralogy of Fallot, atrial or ventricular septal defects, and patent ductus arteriosus.

A small number of patients presented with atypical additional malformations or combined syndromes (see Table 2): A total of 14 patients had abnormalities concerning the gastrointestinal tract with 2 cases of duodenal atresia or stenosis, 1 case of dolichocolon, 2 patients with gallbladder agenesis, 6 cases of anal atresia (4 with VACTERL association), and 5 cases of esophageal atresia (4 with VACTERL association). Altogether, eight patients showed combined malformations that fit the criteria for VACTERL association (VACTERL-PLUS) proposed by van de Putte et al. [38] (see Table 3). 

The first patient had anal and esophageal atresia as well as unilateral renal agenesis and block vertebrae. The second case had a ureteral stricture with megaureter, malformations of the spine and ribs including block vertebrae as well as anal and esophageal atresia. The third case presented with unilateral renal agenesis and a contralateral pelvic kidney, scoliosis, a malformation of the radial ray, esophageal atresia, and single-sided deafness. The fourth case had renal agenesis, thumb hypoplasia, scoliosis, omphalocele, unilateral mamma hypoplasia, and a double-chambered right ventricle as well as a ventricular septum defect (VSD). Case number five had anal and esophageal atresia, thumb aplasia, a VSD, a pelvic kidney, and unspecified vertebral defects. The sixth patient showed a combination of anal atresia, block vertebrae with scoliosis, as well as a multicystic dysplastic kidney. The seventh patient presented with a horseshoe kidney, a VSD, and scoliosis. Case number eight had unilateral renal agenesis, tetralogy of Fallot, as well as scoliosis.

Another patient showed overlapping features of caudal regression syndrome and VACTERL with tethered spinal cord, CTEV, unilateral kidney aplasia, esophageal atresia, and an atrial as well as a ventricular septum defect. 

A TCF2 mutation was found in one patient that led to kidney dysplasia as well as maturity-onset diabetes of the young (MODY), typically summarized as renal cyst and diabetes (RCAD) syndrome or MODY 5. Another patient presented with Silver–Russell syndrome (SRS) and abnormal vertebral segmentation. Furthermore, there were two patients with radial aplasia and thrombocytopenia (TAR syndrome), one patient with agenesis of the pectoralis muscle (Poland syndrome), and another patient with a 22q11 microdeletion (DiGeorge syndrome), also showing cardiac and skeletal defects.

### Uterine Rudiments 

Of the 315 MRKHS patients who underwent surgery at our department between January 2014 and December 2022, 310 fulfilled the inclusion criteria with sufficient information on the surgical field and the existence of uterine rudiments. Five patients were excluded due to missing information on the presence of uterine rudiments. 

**Figure 1:**
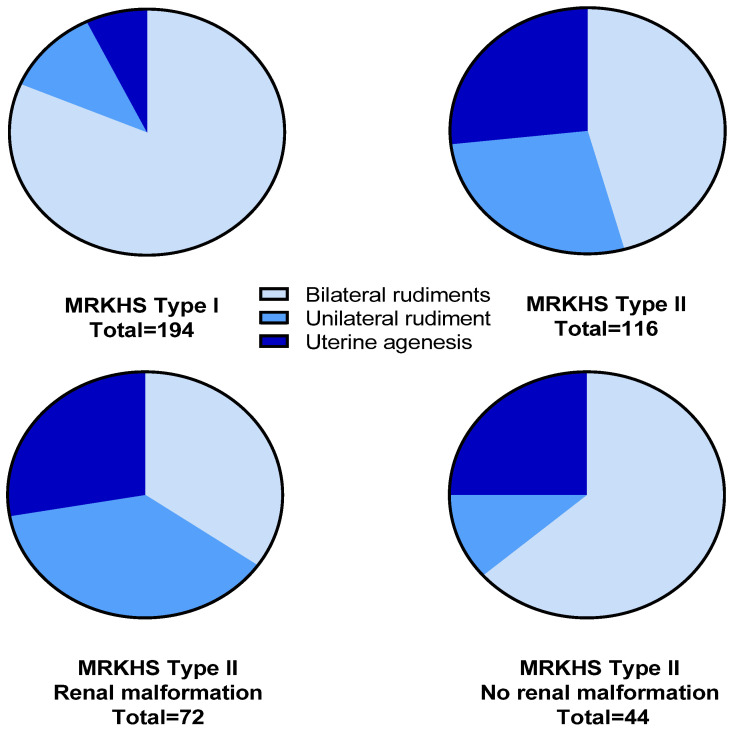
Distribution of uterine rudiments in different MRKHS subtypes. For exact percentages, please see Table 4.

The presence of uterine rudiments was confirmed by laparoscopy in 265 (85.5%) of 310 MRKHS patients (180 MRKHS type I and 85 type II), with 211 patients (68.1%) having bilateral rudiments (see Table 4 and Figure 1). 

The percentage of patients with MRKHS type II and uterine rudiments was lower (73.3%) than of patients with type I (92.8%) (*p* < 0.001). Furthermore, patients with MRKHS type I more frequently had bilateral rudiments with 81.4% compared to only 45.7% for patients with type II (*p* < 0.001). Bilateral rudiments were even less common (34.7%) in patients with renal malformations (*n* = 72). However, bilateral rudiments were more common (63.6%) within patients with associated malformations but no renal anomalies (e.g., only skeletal defects, *n* = 44). 

Of the 45 patients with unilateral kidney agenesis, 12 patients also had uterine agenesis. A total of 26 patients had a unilateral rudiment with 96.2% (25/26) found on the opposite side of renal agenesis and 7 patients presented with bilateral rudiments. In total, only 8 (17.8%) of the 45 patients with unilateral kidney agenesis had a uterine rudiment at the affected side, whilst 32 patients (71.1%) had a contralateral uterine rudiment.

A total of 298 uterine rudiments were removed during surgery from 149 patients with MRKHS type I (76.0%) and 47 patients with MRKHS type II (24.0%). These are described in the following section (see Table 5). A total of 19 rudiments from 11 patients had to be excluded from the analysis due to missing data on rudiment size.

**Figure 2:**
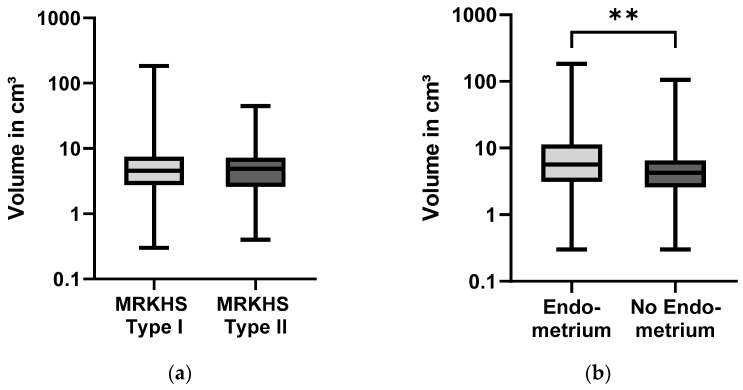
(**a**) Volume of uterine rudiments in MRKHS type I (*n* = 218, mean = 8.4 cm^3^, median = 4.6 cm^3^) and II (*n* = 61, mean = 6.1 cm^3^, median = 4.9 cm^3^). Whiskers mark min and max value; (**b**) Volume of uterine rudiments with (*n* = 89, mean = 13.3 cm^3^, median = 5.6 cm^3^) and without (*n* = 190, mean = 5.4 cm^3^, median = 4.3 cm^3^) endometrial tissue inside. Whiskers mark min and max value (** *p* < 0.010).

Rudiment size ranged from 0.3 cm^3^ to 184.3 cm^3^ with a mean value of 7.9 cm^3^ (*n* = 279). There were no significant differences in rudiment size between MRKHS type I and II (*p* = 0.126). The median for rudiment size was slightly higher in patients with MRKHS type II (*n* = 61, median = 4.9 cm^3^, mean = 6.1 cm^3^, range: 0.4–45.0 cm^3^, SD = 6.6 cm^3^), yet MRKHS type I showed a larger mean size (*n* = 218, median = 4.6 cm^3^, mean = 8.4 cm^3^, range = 0.3–184.3 cm^3^, SD = 18.9 cm^3^) due to some very large rudiments (see Figure 2).

Endometrial tissue was confirmed by histopathological examination in 96 rudiments from 80 patients (40.8% of all patients with rudiment removal). Patients with MRKHS type I were more likely (45.6%) to have endometrium in their rudiments compared to patients with MRKHS type II (25.5%) (*p* = 0.014). In patients with renal malformations (*n* = 27), endometrium was even more seldomly found (22.2%).

Uterine rudiments containing endometrial tissue were significantly larger (mean = 13.3 cm^3^, SD = 27.0 cm^3^) compared to rudiments that did not contain endometrial tissue (mean = 5.4 cm^3^, SD = 8.1 cm^3^) (see Figure 2; *p* = 0.008). Additionally, patients with bilateral rudiments (*n* = 157) more frequently had endometrium (43.3%) than patients with only unilateral rudiments (30.8%, *n* = 39). Out of 102 patients with bilateral rudiment removal, 16 had endometrial tissue in both rudiments (15.7%).

A total of 89 women, who had rudiments removed, had a history of cyclic abdominal pain. Another 10 women reported cyclical complaints but no abdominal pain (e.g., mood swings, headache). However, there was no significant difference in the frequency of endometrial tissue in patients with cyclic abdominal pain (37.1%) compared to patients with no cyclical complaints at all (44.0%, *n* = 84, *p* = 0.351).

Table 6 compares the data of our study with results from other studies on large patient cohorts with MRKHS from China (Pan et al. [13]), Denmark (Herlin et al. [5]), and Germany (Oppelt et al. [7]). 

## 4. Discussion

In this study, we were able to describe the characteristics of a large cohort of MRKHS patients with 469 cases analyzed by a standardized procedure. A total of 35.2% of the patients in our cohort had at least one associated malformation, which resulted in a classification as type II. This rate is slightly lower than reported in the literature so far (43.5–53.0%) for patient cohorts from central Europe [5,7,8,12]. Interestingly, a large cohort study by Pan et al. (2016) that analyzed a Chinese population of 594 patients yielded a very different rate. Only 43 out of 594 (7.2%) patients had associated malformations, with renal anomalies being the most frequent finding [13]. However, another study by Wang et al. (2017) found 46% of their cases to have associated malformations within a Chinese population of 92 MRKHS patients [41]. This difference vividly illustrates the heterogeneity of the MRKHS, thereby highlighting the importance of further investigation regarding geographic differences in phenotypes of MRKHS patients as well as necessary further research into causal mechanisms.

Renal anomalies were the most frequent associated malformation in our cohort with 22.8% of all patients being affected. Unilateral renal agenesis was present in 63.6% of all patients with renal malformations, consistent with the rates proposed in the literature [7,8]. Congenital anomalies of the kidneys and urinary tract (CAKUT) normally occur in about 3 to 9 cases per 1000 live births [42,43]. Therefore, renal malformations appear more frequently in patients with MRKHS than in the general population, most likely due to the proximity and interactions between both organ systems during embryonic development [7]. All patients with Müllerian duct anomalies should therefore be assessed for renal malformations and vice versa.

Among our cohort of 469 patients, several patients had atypical associated malformations or combined syndromes. One patient presented with kidney dysplasia and maturity-onset diabetes of the young, both most likely caused by a mutation in the TCF2 (transcription factor 2) gene. TCF2 encodes for the hepatocyte nuclear factor-1 beta (HNF-1β), which is part of the HNF1 homeoprotein family [44,45]. HNF-1β is expressed during the embryonic development of the liver, intestine, pancreatic islets, lungs, and kidneys. Experiments in mice also revealed its expression in the Wolffian and Müllerian ducts, thus hinting at its role in normal genital tract development [45,46]. Mutations in TCF2 can lead to a variation of phenotypes, probably due to its wide expression during fetal development. Renal disease is the most consistent feature and includes renal cysts, familial hypoplastic glomerulocystic kidney disease, and renal malformations. Mutations in TCF2 can also lead to the autosomal dominant, young onset subtype of diabetes called MODY [47], with the combination being classified as renal cyst and diabetes syndrome (RCAD). Genital tract abnormalities associated with TCF2 mutations include atresia of vas deferens, asthenospermia, uterus didelphys, and bicornuate uterus, as well as aplasia of the uterus and vagina/MRKHS [44,47,48,49,50]. However, two previous studies could not find damaging mutations in the TCF2 gene in a total of 76 patients with MRKHS [15,17]. RCAD should, nevertheless, be kept in mind in patients with MRKHS and renal disease, especially in combination with diabetes.

Another patient had a rare combination of MRKHS and Silver–Russell syndrome (SRS). SRS is an imprinting disorder that leads to prenatal and postnatal growth reduction, relative macrocephaly, a triangular face with a prominent forehead, and asymmetry of the body, as well as feeding difficulties [51,52]. The most common causal mechanisms are a loss of methylation on chromosome 11p15 affecting the Imprinting Center Region 1 (ICR1) (30–60% of all patients) or maternal uniparental disomy of chromosome 7 (5–10% of all patients) [51,53,54]. The co-occurrence of MRKHS and SRS has only been reported in a handful of cases so far [54,55,56,57]. Bruce et al. (2009) originally proposed a correlation between the hypomethylation level of 11p15 and phenotype severity, including two female SRS patients with extreme hypomethylation as well as MRKHS [55]. Abraham et al., (2014), however, reported on a case with MRKHS and SRS but normal methylation of 11p15 (clinical diagnosis of SRS), raising the possibility of unknown common molecular mechanisms that lead to the co-occurrence of MRKHS and SRS [57]. Eggermann et al., (2018) screened 105 MRKHS patients for aberrant methylation at several imprinted loci, including 11p15, yet no imprinting defects were found. This suggests that methylation patterns at presently known disease-causing imprinted loci are not relevant for the MRKHS phenotype [54].

The VACTERL association (OMIM 192350) describes a variable combination of birth defects including vertebral defects, anal atresia, cardiac defects, tracheoesophageal fistula/esophageal atresia, renal anomalies, and limb abnormalities. Typically, the co-occurrence of three or more of the aforementioned defects is required for diagnosis. However, there is an ongoing debate regarding the diagnostic criteria for establishing VACTERL. In an attempt to meet the unmet need, van de Putte et al. (2019) provided a detailed list of congenital malformations that should be counted towards the VACTERL components [38], which we adapted for our study. Applying these rules, 8 of our 469 MRKHS patients qualified for the diagnosis of VACTERL-PLUS. One more patient in our cohort had received a VACTERL diagnosis beforehand due to having anal atresia and tetralogy of Fallot, as well as right-sided lung dysplasia and hypoplasia. Nevertheless, she did not fulfill the criteria proposed by van de Putte et al. (2019) [38], which highlights the need for clear diagnostic criteria for establishing a VACTERL diagnosis.

VACTERL association with concomitant MRKHS has only been rarely reported in the literature [8,58,59,60,61,62,63,64,65]. Cases up to 2016 have been summarized by Bjørsum-Meyer et al. (2016), with anorectal malformations (ARM) and renal anomalies as common characteristics in all nine patients [59]. A recent study by Yano et al. described another nine cases from Japan, all of whom also had ARM [66]. Interestingly, anorectal malformation was only present in four subjects, whilst renal defects were present in every single one of our eight patients with MRKHS and VACTERL. ARM is typically observed in 55–83% of all patients with VACTERL association [38,67,68]. Whilst the majority of VACTERL cases are sporadic, reports of familial occurrence, as well as single component features appearing more frequently in relatives of affected individuals, indicate that genetic factors play a role [69,70,71]. However, similar to the MRKHS, mutations and copy number variations of candidate genes have only been found in a small number of patients [59,72,73,74]. Therefore, clinical diagnosis is necessary in most cases. The co-occurrence of VACTERL and MRKHS could hint toward shared molecular pathways being affected that are crucial for the correct development of organ systems. Due to the embryologic ties between the (uro)genital tract, meso- and metanephros, as well as the anorectal system, an initial defect with subsequent disruption of the development of other organ systems seems possible as well [59,75]. In line with this assumption, renal and/or anorectal malformations were present in every patient with VACTERL and MRKHS in our study cohort. Further research is necessary to understand the causal mechanisms leading to the complex combination of these malformations.

In this study, 68.1% of the patients who underwent laparoscopy at our department presented with bilateral uterine rudiments, 17.4% had a unilateral rudiment, and 14.5% showed complete agenesis of the uterus. This is consistent with a study by Oppelt et al. (2006) that reported bilateral agenesis in 23%, a unilateral rudiment in 4%, and bilateral uterine rudiments in 74% of the cases [12]. Pompili et al. (2009) found bilateral agenesis in 4.9%, a unilateral remnant in 19.5%, and bilateral rudiments in 75.6% of their patients during laparoscopy [76]. Wang et al. (2017) described that 22% of their cases showed uterine agenesis, 10% a unilateral rudiment, and 68% bilateral uterine rudiments during MRI [41]. A total of 585 out of the 594 Chinese MRKHS patients analyzed by Pan et al. (2016) using the VCUAM classification showed stage 4b uterine development. However, the VCUAM classification does not differentiate between bilateral rudimentary or aplastic uterine development, which leaves the exact number of patients with bilateral rudiments undefined [13]. Therefore, it remains unclear if the significantly lower rate of associated malformations in this cohort was accompanied by a different frequency of rudiments as well.

Unfortunately, there are little data available concerning the average size of uterine rudiments in MRKHS patients, mostly through MRI studies. The average volume of a rudiment in our study was 7.9 cm^3^, measured during examination in pathology. Hall-Craggs et al. (2013) examined a total of 115 uterine rudiments through MRI, with the average volume of a uterine rudiment being 6.4 cm^3^ [77].

Renal malformations, especially unilateral renal agenesis, had a noticeable impact on the frequency of uterine rudiments in our patients. A total of 57.8% of patients with kidney agenesis only had a unilateral uterine rudiment, with 96.2% contralateral to the side of the missing kidney. In line with our findings, Preibsch et al. (2014), who examined MRI scans of MRKHS patients who presented at our clinic between 2002 and 2012, reported that 57% of their cases with unilateral renal agenesis only had unilateral rudiments, of which 83% were found at the side of the functioning kidney [78].

Furthermore, patients with renal malformations had the lowest frequency of endometrial tissue (22.2%) in our study. The Wolffian (mesonephric) duct is necessary for regular kidney development by forming the ureteric buds that invade the nearby metanephric mesenchyme [79]. At the same time, the Wolffian duct plays a crucial role in the caudal elongation of the Müllerian duct, inter alia by paracrine Wnt9b-signaling [80,81]. It seems likely that the causal mechanism for kidney agenesis in these patients also affected the elongation of the Müllerian duct, halting development at an earlier stage than usual in MRKHS without forming a uterine rudiment at the affected side.

Overall, endometrial tissue was seen in 40.8% of all patients who had rudiments removed. A similar rate was reported by Rall et al. (2013), who found endometrial tissue in 45.2% of their cases [6]. Larger rudiments were more likely to contain endometrial tissue in our study, most likely due to further advanced uterine development in these patients. However, the presence of endometrium is clearly not solely responsible for the cyclic abdominal pain that many patients experience. Since there was no significant difference regarding the frequency of endometrial tissue in asymptomatic patients (44.0%) compared to patients with cyclic abdominal pain (37.1%), other factors such as endometriosis or mittelschmerz (ovulation pain) must be considered as well.

As described above, our data are based on a single-center study with patients predominantly from central Europe. As mentioned before, recent studies hint at geographic discrepancies between patient cohorts which must be kept in mind regarding the applicability of our findings. Moreover, the reported frequency of endometrial tissue in our study might underestimate the real frequency of endometrial tissue due to a possible sampling error during the histopathological workup as well as preceding removal of tissue for scientific research.

## 5. Conclusions

We analyzed a large cohort of 469 MRKHS patients and observed at least one associated malformation in a third of all subjects. Several patients had atypical malformations or combined syndromes, yet it remains unclear if this concomitance is purely by chance or due to common etiological mechanisms. The range of malformations associated with the MRKHS/MURCS spectrum, however, is vast, thereby demanding close attention regarding additional malformations in other organ systems during clinical evaluation of patients with genital defects.

This study is one of the first to analyze a larger number of MRKHS patients regarding the presence of uterine rudiments as well as their characteristics and indicates a heterogeneity between MRKHS type I and II. Thus, we show that certain variables such as MRKHS subtype or concomitant malformations are associated with a different frequency of uterine tissue, rudiment size, and incidence of endometrial tissue, thereby highlighting the need for further investigation into the responsible associated molecular pathways and potential differences between MRKHS subtypes. Additionally, the results from studies on MRKHS patients from other ethnic groups mark the importance of future research regarding geographical and ethnic discrepancies as well as possible varying causal mechanisms.

## Figures and Tables

**Table 1:** Typical associated malformations of 165 patients with MRKHS type II. More than one malformation per patient is possible.

Typical Associated Malformations	Number	% of Subgroup	% of Type II	% of All Patients
Renal malformations	107		64.8%	22.8%
Unilateral renal agenesis	68	63.6%	41.2%	14.5%
Pelvic kidney	18	16.8%	10.9%	3.8%
Duplex kidney	14	13.1%	8.5%	3.0%
Malrotated kidney	9	8.4%	5.5%	1.9%
Horseshoe kidney	6	5.6%	3.6%	1.3%
Other renal malformations	11	10.3%	6.7%	2.3%
Skeletal malformations	81		49.1%	17.3%
Scoliosis	45	55.6%	27.3%	9.6%
Block vertebrae	10	12.3%	6.1%	2.1%
thereof Klippel–Feil	5	6.2%	3.0%	1.1%
Polydactyly	5	6.2%	3.0%	1.1%
Syndactyly	3	3.7%	1.8%	0.6%
CTEV	3	3.7%	1.8%	0.6%
Hip dysplasia	8	9.9%	4.8%	1.7%
Other associated malformations				
Auditory	6		3.6%	1.3%
Ocular	2		1.2%	0.4%
Cardiac	18		10.9%	3.8%

**Table 2:** Selected atypical associated malformations and combined syndromes.

Atypical Associated Malformations	Number
Gastrointestinal tract anomalies	14
Duodenal atresia or stenosis	2
Dolichocolon	1
Anal atresia	6
Esophageal atresia	5
Gallbladder agenesis	2
VACTERL association	8
TAR syndrome	2
Poland syndrome	1
DiGeorge syndrome	1
RCAD syndrome	1
Silver–Russell syndrome	1

**Table 3:** Characteristics of the eight patients with MRKHS and VACTERL association. For a full list of which congenital malformations are counted as major or minor VACTERL features, please see van de Putte et al., (2019) [38].

Patient	Vertebral	Anal	Cardial	Tracheo/ Esophageal	Renal	Limbs
1	Block vertebrae	Anal atresia	-	Esophageal atresia	Unilateral renal agenesis	-
2	Block vertebrae	Anal atresia	-	Esophageal atresia	Megaureter	-
3	Scoliosis	-	-	Esophageal atresia	Unilateral renal agenesis, Pelvic kidney	Radial-ray malformation
4	Scoliosis	-	Double-chambered right ventricle, VSD	-	Unilateral renal agenesis	Thumb hypoplasia
5	Unspecified vertebral defects	Anal atresia	VSD	Esophageal atresia	Pelvic kidney	Thumb aplasia
6	Block vertebrae, Scoliosis	Anal atresia	-	-	Multicystic dysplastic kidney	-
7	Scoliosis	-	VSD	-	Horseshoe kidney	-
8	Scoliosis	-	Tetralogy of Fallot	-	Unilateral renal agenesis	-

**Table 4:** Patient characteristics and frequency of uterine rudiments.

	Type I	Type II	Type II	Type II
			Renal malformation	No renal malformation
Number	194	116	72	44
Bilateral rudiments	158 (81.4%)	53 (45.7%)	25 (34.7%)	28 (63.6%)
Unilateral rudiment	22 (11.3%)	32 (27.6%)	27 (37.5%)	5 (11.4%)
Uterine agenesis	14 (7.2%)	31 (26.7%)	20 (27.8%)	11 (25.0%)

**Table 5:** Characteristics of patients who had rudiments removed during surgery, respective mean rudiment volume, and endometrial tissue frequency of patients. A total of 19 rudiments from 11 patients had to be excluded due to incomplete measurement.

	Type I	Type II	Type II	Type II
			Renal malformation	No renal malformation
Number of patients (included/total rudiments)	149 (218/235)	47 (61/63)	27 (33/34)	20 (28/29)
Mean size in cm^3^ (range)	8.4 (0.3–184.3)	6.1 (0.4–45.0)	7.3 (0.5–45.0)	4.7 (0.4–11.9)
Endometrium	45.6%	25.5%	22.2%	30.0%

**Table 6:** Comparison of results from studies on large MRKHS patient cohorts from different countries (Germany, Denmark, and China). Percentages are based on the number of patients for whom the respective characteristic was recorded. Variables were marked as not available (NA) if they were either not recorded in the respective study or not reported.

	Oppelt et al. [7]	Herlin et al. [5]	Pan et al. [13]	This study
Setting	Two center study	National patient registry	Single center study	Single center study
Number of patients	284	168	594	469
Associated malformations	45.1%	43.5%	7.2%	35.2%
Renal malformations	29.6%	34.2%	5.2%	22.8%
Kidney agenesis	18.7%	21.6%	3.9%	14.5%
Pelvic kidney	9.2%	6.3%	1.0%	3.8%
Skeletal malformations	19.0%	12.5%	2.0%	17.3%
Scoliosis	NA	NA	1.2%	9.6%
Patients undergoing laparoscopy	284	NA	594	310
Distribution of uterine development	84.2% bilateral/9.5% unilateral rudimentary or aplastic	NA	98.5% bilateral/0.5% unilateral rudimentary or aplastic	68.1% bilateral/ 17.4% unilateral rudiment
Mean rudiment size	NA	NA	NA	7.9 cm^3^
Endometrial tissue	NA	NA	NA	40.8%

## Data Availability

The data presented in this study are available on request from the corresponding author. The data are not publicly available due to patient privacy policy.
